# Complete genome sequence of hydroquinonesulfonate-assimilating bacterium, *Delftia lacustris* HQS1

**DOI:** 10.1128/mra.00371-24

**Published:** 2024-12-27

**Authors:** Masahiro Takeo, Seiwa Ohtaki, Hidehiro Ishizawa

**Affiliations:** 1 Department of Applied Chemistry, Graduate School of Engineering, University of Hyogo, Himeji, Hyogo, Japan; California State University San Marcos, San Marcos, USA

**Keywords:** biodegradation, aromatic sulfonates, hydroquinonesulfonate, phenolsulfonate, bioremediation

## Abstract

We report the complete genome assembly of a hydroquinonesulfonate-assimilating bacterium, *Delftia lacustris* strain HQS1. This strain contains one circular chromosome (6,979,964 bp) and one circular plasmid (39,999 bp). The chromosomal sequence contained 6,359 coding sequences and a gene cluster involved in the degradation of gentisate, which is structurally similar to hydroquinonesulfonate.

## ANNOUNCEMENT

**Fig 1:**
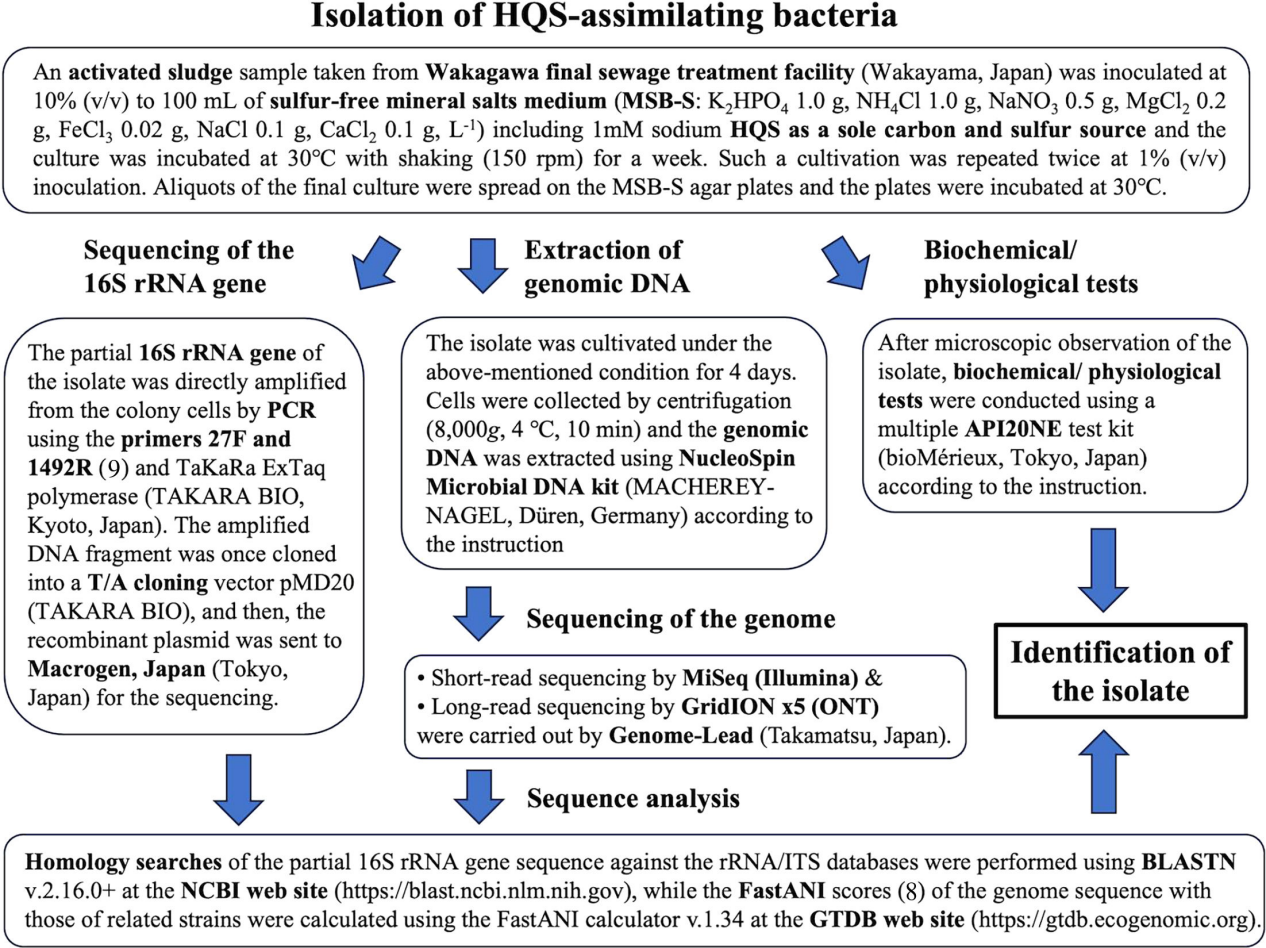
Summary of the isolation and identification of the HQS-assimilating bacterium, strain HQS1 (8, 9).

Aromatic sulfonates (ASs) have been produced as industrial products, such as dyes and surfactants, and released into aquatic environments through various applications (1). ASs are generally recalcitrant to biodegradation, and thus, they form one category of the major pollutants in wastewaters and natural waters (1–3). Attempts to remove ASs from polluted waters by photocatalysis and ozonation have been conducted (4–6). However, such processes accumulate hydroxylated aromatic intermediates such as hydroquinonesulfonate (HQS) (4–6). To investigate the possibility of their biodegradation, we isolated an HQS-assimilating bacterium from activated sludge and named it strain HQS1 in July 2018. Figure 1 summarizes the isolation and identification of strain HQS1. The partial 16S rRNA gene sequence (1,383 bp) showed 99.93% and 99.86% identities with those of *Delftia lacustris* 332^T^ (NR_116495) and *Delftia tsuruhatensis* T7^T^ (NR_024786), respectively. Strain HQS1 showed no growth at >35°C and assimilation positives for D-mannitol and DL-malate, suggesting that it belongs to *D. lacustris*, based on Jørgensen et al.’s descriptions (7).

Genomic DNA was extracted from the HQS1 cells (Fig. 1) and used for short-read and long-read sequencing. For short-read sequencing, the paired-end library was prepared using a Nextera DNA Flex Library Prep Kit (Illumina, San Diego, USA) and was sequenced (at 159 bp cycles) on an MiSeq instrument (Illumina), generating 2,526,506 paired reads (0.39 Gb, average 154 bp). For long-read sequencing, DNA fragments less than 10 kb were first removed using a Short Read Eliminator XS kit (PacBio, California, USA) (without shearing process), and then, another DNA library was prepared using a Ligation Sequencing Kit (Oxford Nanopore Technologies [ONT]). The prepared library was applied to a FLO-MIN106 R9.41 flow cell on a GridION X5 sequencer (ONT). The long-read sequences, which were base-called using Guppy v.4.2.3, generated 209,120 reads (1.9 Gb, average 9.2 kb, *N*_50_ = 15.0 kb) during a 24 h runtime. Low-quality short reads with average *Q* values of >30.0 and the adapter sequences were trimmed with fastp v.0.20.1 (10), resulting in short-read sequences (0.36 Gb, average 150 bp). Low-quality nanopore reads with average *Q* values of >10.0 and the adapter sequences were trimmed with NanoFilt v.2.7.1 (11), resulting in long-read sequences (1.4 Gb, average 10.7 kb). Unicycler v.0.4.8 (12) was used for *de novo* genome assembly, followed by a polishing step with Pilon v.1.23 (13). The resulting scaffolds were analyzed by Bandage v.0.8.1 (14), confirming the circularity and generating a single circular contig for the chromosome with a length of 6,979,964 bp (G + C: 66.5%) and another circular contig for a plasmid with a length of 39,999 bp (G + C: 62.3%). Annotation was carried out using DFAST v.1.2.4 (15), which predicted 6,359 coding sequences in the chromosome as well as 15 rRNA and 83 tRNA genes. A gene cluster involved in the degradation of gentisate, which is structurally similar to HQS, was also found at around 3,309 kb in the sequence.

By the FastANI analysis (Fig. 1) (8), the genome sequence of strain HQS1 showed extremely high scores with those of *D. lacustris* LzhVag01 (NZ_CP141536) (99.90%), *D. lacustris* 332^T^ (98.18%), and *D. tsuruhatensis* T7^T^ (98.38%), supporting the above-mentioned identification.

## Data Availability

The partial 16S rRNA gene, genome, and plasmid sequences of strain HQS1 have been deposited in DNA Data Bank of Japan (DDBJ) under accession nos. LC823183, AP025556, and AP025557, respectively. Raw sequencing data were deposited in the DDBJ Sequence Read Archive (SRA) database under BioProject no. PRJDB12370 and BioSample no. SAMD00407106. The SRA accession nos. are DRX309261 (ONT) and DRX309260 (Illumina).

## References

[1] Schwitzguébel JP, Aubert S, Grosse W, Laturnus F. 2002. Sulphonated aromatic pollutants. Limits of microbial degradability and potential of phytoremediation. Environ Sci Pollut Res Int 9:62–72. doi:10.1007/BF02987317 11885419

[2] Alonso MC, Castillo M, Barceló D. 1999. Solid-phase extraction procedure of polar benzene- and naphthalenesulfonates in industrial effluents followed by unequivocal determination with ion-pair chromatography/electrospray-mass spectrometry. Anal Chem 71:2586–2593. doi:10.1021/ac981377e 21662805

[3] Terzić S, Senta I, Ahel M, Gros M, Petrović M, Barcelo D, Müller J, Knepper T, Martí I, Ventura F, Jovancić P, Jabucar D. 2008. Occurrence and fate of emerging wastewater contaminants in Western Balkan Region. Sci Total Environ 399:66–77. doi:10.1016/j.scitotenv.2008.03.003 18420255

[4] Brezová V, Jankovičová M, Soldán M, Blažková A, Reháková M, Šurina I, Čeppan M, Havlínová B. 1994. Photocatalytic degradation of p-toluenesulphonic acid in aqueous systems containing powdered and immobilized titanium dioxide. J Photochem Photobiol A: Chem 83:69–75. doi:10.1016/1010-6030(94)03804-X

[5] Szabó-Bárdos E, Markovics O, Horváth O, Töro N, Kiss G. 2011. Photocatalytic degradation of benzenesulfonate on colloidal titanium dioxide. Water Res 45:1617–1628. doi:10.1016/j.watres.2010.11.045 21185053

[6] Zsilák Z, Fónagy O, Szabó-Bárdos E, Horváth O, Horváth K, Hajós P. 2014. Degradation of industrial surfactants by photocatalysis combined with ozonation. Environ Sci Pollut Res 21:11126–11134. doi:10.1007/s11356-014-2527-2 24448882

[7] Jørgensen NOG, Brandt KK, Nybroe O, Hansen M. 2009. Delftia lacustris sp. nov., a peptidoglycan-degrading bacterium from fresh water, and emended description of Delftia tsuruhatensis as a peptidoglycan-degrading bacterium. Int J Syst Evol Microbiol 59:2195–2199. doi:10.1099/ijs.0.008375-0 19605727

[8] Jain C, Rodriguez-R LM, Phillippy AM, Konstantinidis KT, Aluru S. 2018. High throughput ANI analysis of 90K prokaryotic genomes reveals clear species boundaries. Nat Commun 9:5114. doi:10.1038/s41467-018-07641-9 30504855 PMC6269478

[9] Rochell PA, Will JAK, Fry JC, Jenkins GJS, Parks RJ, Turley CM, Weightman AJ. 1995. Extraction and amplification of 16S rRNA genes from deep marine sediments and seawater to assess bacterial community diversity. In Trevors JT, van Elsas JD (ed), Nucleic acids in the environment. Springer-Verlag, Berlin, Germany.

[10] Chen S, Zhou Y, Chen Y, Gu J. 2018. fastp: an ultra-fast all-in-one FASTQ preprocessor. Bioinformatics 34:i884–i890. doi:10.1093/bioinformatics/bty560 30423086 PMC6129281

[11] De Coster W, D’Hert S, Schultz DT, Cruts M, Van Broeckhoven C. 2018. NanoPack: visualizing and processing long-read sequencing data. Bioinformatics 34:2666–2669. doi:10.1093/bioinformatics/bty149 29547981 PMC6061794

[12] Wick RR, Judd LM, Gorrie CL, Holt KE. 2017. Unicycler: resolving bacterial genome assemblies from short and long sequencing reads. PLoS Comput Biol 13:e1005595. doi:10.1371/journal.pcbi.1005595 28594827 PMC5481147

[13] Walker BJ, Abeel T, Shea T, Priest M, Abouelliel A, Sakthikumar S, Cuomo CA, Zeng Q, Wortman J, Young SK, Earl AM. 2014. Pilon: an integrated tool for comprehensive microbial variant detection and genome assembly improvement. PLoS One 9:e112963. doi:10.1371/journal.pone.0112963 25409509 PMC4237348

[14] Wick RR, Schultz MB, Zobel J, Holt KE. 2015. Bandage: interactive visualization of de novo genome assemblies. Bioinformatics 31:3350–3352. doi:10.1093/bioinformatics/btv383 26099265 PMC4595904

[15] Tanizawa Y, Fujisawa T, Nakamura Y. 2018. DFAST: a flexible prokaryotic genome annotation pipeline for faster genome publication. Bioinformatics 34:1037–1039. doi:10.1093/bioinformatics/btx713 29106469 PMC5860143

